# The development of EFL Learners’ willingness to communicate and self-efficacy: The role of flipped learning approach with the use of social media

**DOI:** 10.3389/fpsyg.2022.1001283

**Published:** 2022-10-26

**Authors:** Xiangping Fan

**Affiliations:** School of Languages and Cultures, Shanghai University of Political Science and Law, Shanghai, China

**Keywords:** flipped learning approach, self-efficacy, social media, willingness to communicate, EFL learners

## Abstract

Promoting English as a Foreign Language (EEL) learners’ willingness to communicate and self-efficacy in different contexts has drawn the attention of many investigators. This review explored the effect of digital-based flipped learning classrooms on enhancing learners’ willingness to communicate and self-efficacy. The related literature indicated that learners’ intention to communicate is affected by social media and digitalized materials used in flipped classrooms. Compared to the traditional educational contexts, this review showed higher levels of self-efficacy in flipped classrooms among EFL learners. Furthermore, the review expounded on the implications and future directions for EFL teachers, teacher educators, educational policy-makers, and advisors. The ideas can improve their awareness of learner self-efficacy, willingness to communicate, and the use of flipped learning approach in educational contexts.

## Introduction

One of the developing approaches that has arisen in the field of education with the utilization of technology and has been generally employed in the last decade is the flipped learning approach. The flipped learning approach can be characterized as a pedagogical approach in which direct instruction moves from the group learning space to the individual learning space, and the resulting group space is transformed into a dynamic, interactive learning environment where the educator guides students as they apply concepts and engage creatively in the subject matter (O’Flaherty and Phillips, 2015). Moreover, flipped learning approach has four main pillars, which comprise the four letters of the word “FLIP.” These pillars are “flexible environment,” “learning culture,” “intentional content,” and “professional educators,” which stand for the four letters of the word “FLIP” (Hu, 2018, p. 5).

Nowadays, as digital communication in flipped learning approach is becoming crucial in everyday life, computer-assisted language learning investigators have sought to understand learners’ language use in digital settings in flipped learning classrooms (Lee and Drajati, 2019; Aghaei et al., 2020). Learners’ willingness to communicate seems to be affected by the flipped learning approach. The idea of willingness to communicate in second-language acquisition is that those language learners who are eager to communicate in the foreign language actively look for opportunities to impart and communicate. Hence, “the ultimate goal of the learning process should be to engender in language education students the willingness to seek out communication opportunities and the willingness actually to communicate in them” (MacIntyre et al., 1998, p. 547). The existing willingness to communicate in first and foreign language studies appears to have focused mostly on non-digital situations (in-class and out-of-class settings), but relatively little on digital settings (Lee and Hsieh, 2019).

Flipped classrooms have had an important role in developing self-efficacy, helping learners form positive attitudes and emotions, and leading to greater learner satisfaction (Abe et al., 2021). Self-efficacy, which refers to learners’ capability to complete a task and the confidence in their skills to perform that task (Pintrich, 1999), is a vital component of learners’ aptitude, achievements, and performance (Bandura, 1997), and is significantly correlated with learners’ earlier learning experiences (Yeh et al., 2019). Although earlier investigations on educational psychology and second language acquisition have highlighted the significance of self-efficacy in traditional educational contexts, recent studies underscored learners’ self-efficacy and its relationship with flipped English classrooms (Namaziandost and Çakmak, 2020).

Bridging the gap between the psychology of language learning and the use of technology, this reviewed the studies on the significance of flipped learning in the development of learners’ self-efficacy and willingness to communicate through using social media. The innovation of this review is to illuminate the relationship between technology and positive emotional construct, which may help educators and learners to develop an appropriate method in educational contexts. Moreover, it provides some ideas for educators and teacher trainees to develop learners’ self-efficacy and willingness to communicate in foreign language. The findings and implications of the present study will provide relatively novel insights for teachers. Exploration in this field can help educators in many aspects of the classroom to find new approaches to become more effective teachers and accordingly make practical learning setting for increasing learners’ proficiency in language use and their self-efficacy. Most of researchers have recognized the need for alternative teaching approaches to meet different students’ requirements in the context of classrooms. This review is significant because it demonstrates the importance of using such a technique, flipped teaching, to improve learners’ willingness to communicate and their self-efficacy.

## Literature review

### Flipped learning approach

For decades, English language investigators have been on the trail of finding an effective and efficient language teaching methodology to foster foreign language skills, consider the changing requirements of learners, and encourage learners to employ more cooperative and individual activities in and outside the classroom (Aghaei and Gouglani, 2016; Chuang et al., 2018). One of the most important instructional approaches to boost language learning is flipped classroom approach. Caner (2012) defined flipped learning as a type of blended learning which is “the blend or mixture of any two instructional technologies” (p. 24). Lage et al. (2000) pointed out that in flipped learning approach, “events that have traditionally taken place inside the classroom now take place outside the classroom and vice versa” (p. 32). Afrilyasanti et al. (2017) defined flipped classroom approach as a type of approach in which learners both take part in-class activities, and they also cope with their online self-learning. They also stated that learners have opportunities to increase their language proficiency at home, and they can involve in activities and collaborate with other learners in the educational contexts. Hung (2018) defined pre-class self-learning as the bringing of linguistic knowledge to learners’ private space and time with the help of videos and related exercises. He also described the in-class activities as pair or group activities that are done during class time. Therefore, the shift of material consignment to the outside of the class and using the class time for higher-level activities like applying and examining the earlier learned materials are the primary components of flipped learning approach (Yilmaz and Baydas, 2017). The flipped learning approach is an educational approach that inverts the process of the conventional classroom by presenting the educational substances typically online, involving the students in cooperative group learning, or potentially basic critical thinking exercises completed under the educator’s direction amid class (Namaziandost and Çakmak, 2020). This approach can help teachers and learners to prevail over some limits in online education, particularly in terms of time, space, and materials constraints (Haghighi et al., 2019). Kawinkoonlasate (2019) also argued that the flipped learning approach, compared to explicit instructional techniques, can integrate learning activities such as role-plays, discussions, and problem-solving activities with learning materials outside the classroom. Erbil (2020) also stated that teachers, in flipped classrooms, employ collaborative techniques that directly involve students by integrating instructions, aspects of the learning background, content, and activities.

Constructivism can be called a theoretical foundation of flipped learning approach. According to Aljohani (2017), constructivism is based on the premise that foreign language learning can be developed by providing the means and the time for learners to engage in classrooms. Li et al. (2021) also stated that, in flipped learning approach, learners can construct their foreign language knowledge in the educational contexts, following the comprehension of rudimentary notions through watching the videos, listening to the audio, and reading the materials at home. The cognitive load theory can also specify the importance of flipped learning approach. Using cognitive load theory, Li (2022) stated that flipped learning classrooms prevent working memory overload, since learners work at their own pace in the pre-class preparation stage. Therefore, he asserted that flipped learning approach provides favorable conditions for language learning.

Vygotsky’s theory of mediation in digital learning environment can be regarded as a theory which relates technology to psychological states. According to Zidoun et al. (2019), education programs should consider the role and impact of technological developments on learning. The concept of technological mediation, inspired by Vygotsky’s (1986) theory of tool mediation, aims to gain insight in the ways in which technology actively co-shapes the relation between people and the world through various mediating effects. De Boer et al. (2018) explain that this understanding of technological mediation emphasizes “the primacy of the relatedness between emotional states of people, technologies, and the world” (p. 300).

### The notion of willingness to communicate

There are so many language learners who do not tend to enter L2 communication situations, despite their high proficiency scores on different language learning tests. This issue proves that there is another construct that intercedes between the competence to communicate and to place this competence into practice (Dörnyei, 2005). This construct is called willingness to communicate. The notion of willingness to communicate was presented in the foreign language learning literature by MacIntyre et al. (1998). They defined this notion as “a readiness to enter the discourse at a particular time with a specific person or persons, using an L2” (p. 547). MacIntyre and Charos (1996) also defined willingness to communicate as “a stable predisposition toward communication when free to choose to do so” (p. 7). Kurk (2019) also mentioned that willingness to communicate refers to a learner’s state of cognitive planning to apply the target language in his communication. MacIntyre and Vincze (2017) considered willingness to communicate as the main objective of foreign language learning since the intention to communicate can result in authentic communication behavior, which leads to an increase in foreign language proficiency. Öz et al. (2015) believed that willingness to communicate is indeed a multi-faceted construct that integrates affective, social-psychological, linguistic, and communicative variables and can describe, explain, and predict language learners’ communicative behavior in an L2.

According to MacIntyre et al.’s (1998) model, foreign language learners’ willingness to communicate has been investigated from trait-like and dynamic, and situated dimensions (Dewaele and Dewaele, 2018). The trait-like or psychological dimension of willingness to communicate is correlated with foreign language anxiety (Liu, 2018), self-confidence, and motivation (Lee and Hsieh, 2019). On the other hand, the dynamic and situated dimensions of willingness to communicate refer to the social and contextual features of education, including interlocutors (Fadilah, 2018), themes of interaction (Mystkowska-Wiertelak and Pawlak, 2016), instructors (Zarei et al., 2019) and cooperative peers (Khajavy et al., 2016). The concept of willingness to communicate has recently drawn the attention of many investigators. Since the establishment of the theory, scholars have begun investigating how willingness to communicate can be directly or indirectly affected by individual factors such as gender (Lee et al., 2021), age (Yetkin and Zekiye, 2022), foreign language anxiety (Kruk, 2022), and language learning motivation (Dewaele and Dewaele, 2018). Concurrently, investigators have begun to realize that willingness to communicate can be focused on dynamic variables such as the role of internet-based resources (Nugroho, 2021).

### The role of flipped learning approach in developing learners’ willingness to communicate through social media

Flipped learning approach, as a wide-ranging model, enables instructors to foster learners’ educational processes (Bergmann, 2018). Das et al. (2022) found that there was an increase in learners’ emotional and cognitive engagement during the employment of flipped learning approach. Tsai et al. (2020) stated that brainstorming and activities in flipped classroom approach *via* social networking can expand learners’ learning performance and self-learning. Hamid et al. (2015) approved the role of online social networking educational activities in enhancing learners’ performance in flipped learning classrooms. They mentioned that learners can leave comments on a blog or discussion forum and ask for more detailed explanations, add someone as a friend, initiate communication by leaving a message, and collaborate with peers to develop their formal and informal interactions by establishing active communication with their peers. Jafarigohar et al. (2019) investigated the effect of flipped learning approach on learners’ cognitive performance. They used the Telegram as the online platform through which the listening materials, such as Ted-talk videos, were sent before the class, and the speaking activities were done through open discussion forums in the Telegram group. They found that flipped learning classroom with the Telegram, as a social media, improves learners’ listening and speaking skills. They justified their findings through social constructivism, learner-centered learning, active learning, and learner autonomy theories. All these theories indicate that learning builds up by social context and a facilitator. They mentioned that cooperative learning, as another important aspect of social constructivism, occurs in flipped classrooms and class time, and it includes practice activities where students complete the tasks in pairs or groups to foster their performance in a foreign language. They also argued that the structure of learning materials posted through the Telegram had a positive effect on how the learners perceived the learning environment and participated in the learning process. In ESL context, Kasuma (2021) found that incorporating Facebook, as a social media, facilitated the use of preferred learning styles and strategies, which enhanced learners’ learning interest, improved their language abilities, and provided them with the best academic experience. Nugroho and Atmojo (2020) explored EFL learners’ insights and instructional activities of digital learning of English in a flipped classroom. Using a web-based survey and semi-structured interview, they found that learners have a positive attitude toward the use of digital technology to learn English outside the classroom. They mentioned that using social media such as Facebook, Instagram, and YouTube can facilitate the EFL learners’ performance and achievement.

Some studies have shown that learners’ willingness to communicate is significantly affected by the social networks in the flipped learning approach. Lee and Hsieh (2019) investigated Korean and Taiwanese learners’ willingness to communicate in flipped learning classrooms. Their study showed that instructional practice affects a higher willingness to communicate in a foreign language in digital settings. They also mentioned that instructors’ rapport, like encouraging learners to socialize with other English users on social media, promotes learners’ willingness to communicate in flipped classrooms. Mohammadi et al. (2019), in their experimental study, employed collaborative tasks and textbook, and in-class quizzes in both traditional and flipped educational contexts. They found that learners utilizing social networks like Telegram have significantly higher levels of willingness to communicate in a foreign language. Their study showed that theory-based flipped instruction using wide-ranging online interaction through the social network like Telegram encouraged learners to involve in more learning tasks by being active and competent in using the learned grammar, vocabulary, and reading materials for communicative interaction, storytelling, dialog development, class discussion, and group presentations, which in turn lead to the higher levels of willingness to communicate. Khosravani et al. (2020) used videos, audio, and reading online materials through the social network to determine their effects on learners’ autonomy, motivation, and willingness to communicate. They considered the construct of willingness to communicate as a stable construct that is stable across time and situations. However, their study revealed that flipped learning approach did not have significant effects on learners’ willingness to communicate. On the contrary, Lee and Lu (2021), in their study, used extramural English activities on the Internet (e.g., watching YouTube clips in English and chatting with others in English *via* social media). Their study indicated that the extramural digital setting significantly affected learners’ willingness to communicate in a foreign language as a dynamic concept. They also found that EFL learners with a clear L2 self-image are highly motivated to learn and practice English, which can prompt their willingness to communicate in English in the classroom. Nugroho (2021) studied the effect of using informal digital English learning in flipped classrooms on Indonesian EFL learners’ willingness to communicate. His study showed that frequency of informal digital English learning, like social media, and the performing of receptive and productive tasks *via* social networks boost EFL learners’ willingness to communicate. They argued that EFL learners’ engagement in digital learning activities such as watching English videos, reading news online, and posting English content, increases their motivation to communicate in a foreign language. Using a mixed method design, Zarrinabadi et al. (2021) investigated the influence of flipped learning approach on EFL learners’ willingness to communicate. They used the Telegram application to assign learners pre-class activities. Learners were supposed to watch the videos, study short texts, and listen to audio recordings related to the content of each lesson. Their study showed that flipped classroom strategy significantly influenced learners’ willingness to communicate by making language learning enjoyable, increasing motivation, and decreasing language anxiety. They argued that practicing the materials through the social network in flipped educational contexts can promote learners’ self-confidence and reduce their stress and anxiety, leading to an increased willingness to communicate.

Earlier studies have indicated that foreign language anxiety, as a negative emotional state, is an influential variable that affects willingness to communicate. (e.g., Dewaele, 2019; Kruk, 2019; Lee and Drajati, 2019). Moreover, social media can significantly affect learners’ foreign language anxiety (Su and Fatmawati, 2019). Desta et al. (2021) noted that social media helps learners to diminish their level of anxiety in language use. They mentioned that social media, used as a learning tool, improves learners’ English language competency by reducing their level of anxiety. Su and Fatmawati (2019) found that the offered project through Facebook can improve learners’ cognitive and psychological matters when dealing with speaking skill. They believed that social media, like Facebook, can relieve the students’ feeling of anxiety which eventually improve their language use. Sharma (2019) also asserted that social media is regarded as a remedy to lower affective variables, increase confidence, improve communication in L2, increase motivation, reduce anxiety, decrease shyness, and to enhance positive attitudes toward language learning. Sun et al. (2017) indicated that EFL learners are likely to experience shyness or even fear when communicating in English. They mentioned that communicating *via* social-networking sites reduces learners’ anxiety about using their target languages, and helps them connect with other learners of the same target language.

Perceived communicative competence is another predictor of foreign language learners’ willingness to communicate (Elahi Shirvan et al., 2019). Studies have shown that using social media affects learners’ perceived communicative competence (e.g., Boatis et al., 2020; Yekimov et al., 2021; Nelyska, 2022). Morreale et al. (2015) mentioned that the ability of learners to communicate competently is impacted by how they use the newer communication technologies now ubiquitous in their daily lives. They mentioned that learners are willing to forgo the abundant benefits of face-to-face communication, most likely in favor of speed and convenience. They argued that social media brings attentiveness, expressiveness, appropriateness, effectiveness, knowledge, efficiency, and motivation. Moreover, it fosters communicative competence. They also asserted that learners, in their study, perceive themselves to be more competent when they are using social media. Puzanov et al. (2022) mentioned that well-known blogs, podcasting, media objects, wikis, and social bookmarks are the most popular social services that can be used effectively to develop learners’ communicative competence. They mentioned that interactivity in social media, such as Facebook, Instagram, and TikTok are helpful to foster learners’ communicative competence and their intention to communicate in a foreign language.

Studies about willingness to communicate in a foreign language have shown that motivation is a significant predictor of this construct. (Khajavy et al., 2019; Alrabai, 2022; Kruk, 2022). Mulyono and Saskia (2020), in their study, investigated the role of self-confidence, motivation, and anxiety in promoting students’ willingness to communicate in traditional and digital settings. They found motivation as an affective variable that influences learners’ willingness to communicate in both digital and traditional environments. The role of social media in promoting learners’ motivation has been verified in earlier studies. Xodabande (2017) also argued that the proper use of social media can enhance learners’ interests and motivation, facilitate students’ access to target language input, provide them with more interaction opportunities and feedback and also give the instructors the tools they need to organize course content. Dirjal et al. (2020) investigated the possible role of social media, particularly Skype, in promoting and developing learners’ motivation for language use. They found that male and female learners were highly motivated after receiving their instruction *via* Skype device. They argued that social media can foster both internal and external motivation among learners, which provides the primary impetus to initiate L2 learning and later the driving force to sustain the long, often tedious learning.

### The notion of self-efficacy

Emotions, as the primary issues in learners’ foreign language learning (Piniel and Albert, 2018), are investigated in various settings. Self-efficacy, as a positive emotional construct, is defined as “people’s judgments of their capabilities to organize and execute courses of action required to attain designated types of performances” (Bandura, 1986, p. 391). Bandura (1986) asserted that self-efficacious individuals rely on their competence to deal with demanding activities, and carry out the required strategies to be effective in forthcoming situations. Jeong et al. (2021) stated that self-efficacy specifies students’ confidence in arranging their learning process and influences their apprehension of cognitive growth. Schunk and Pajares (2010) also indicated that individuals with higher levels of self-efficacy are inclined to have higher intrinsic interest, set themselves thought-provoking objectives, and keep a strong commitment to activities. Bandura (1997) listed four primary sources of self-efficacy beliefs as (1) enactive mastery experiences, (2) vicarious experiences, (3) verbal persuasion, and (4) the physiological and affective state of an individual. Zhang and Ardasheva (2019) stated that enactive mastery experiences, are the most significant cause of self-efficacy. They mentioned that enactive mastery experiences are related to an individual’s insight over his/her own capability to positively undertake a specific task informed by earlier accomplishments. They mentioned that enactive mastery experiences are related not only to individuals’ perception of their capability, but also the task’s difficulty, and the amount of effort they will exert to accomplish the task. According to Wilde and Hsu (2019), vicarious experiences, as the second source of self-efficacy, are concerned with social comparison of a person’s performance to that of others with similar abilities. El-Abd and Chaaban (2021) asserted that observing others’ comparable capabilities can improve one’s self-efficacy by approving the sufficiency of his/her knowledge, abilities, and approaches. Verbal persuasion, the third source of self-efficacy, refers to “socially persuasive feedback, comments by significant others regarding one’s performance” (Bandura, 1997, p. 20). Wangwongwiroj and Yasri (2021) mentioned that constructive comments emphasizing an individual’s aptitudes or achievements will improve self-efficacy. The physiological and affective state of an individual, the fourth source of self-efficacy, is related to individuals’ capability to control bodily and emotional stress reactions (e.g., breathing, anxiety) over task performance (Webb-Williams, 2018).

### The role of flipped learning approach in developing learners’ self-efficacy

Integrating positive individual learning experiences can lead to increased accomplishment in the educational context (Wagner et al., 2020). Some investigations about the effect of flipped learning approach on learner self-efficacy have drawn the attention of many scholars (e.g., Iyitoğlu and Erişen, 2017; Doo and Bonk, 2020; Namaziandost et al., 2020). The investigations on self-efficacy and flipped classes underscored positive emotional states, since self-efficacy stems from meeting basic cognitive needs such as a sense of competence, autonomy, and social interaction (Ha et al., 2019). In studying the relationship between web-based flipped learning and self-efficacy, Su Ping et al. (2020) examined the variables in which learners demonstrated improvement after flipped classroom learning. To create more opportunities for interactive class activities and discussions in flipped learning approach, they converted two-thirds of the course content into 115 min of web-based conferences. They recorded their interaction using the accessible version of Camtasia. Using semi-structured interviews, they found that learners’ practice, commitment, communicative competence, motivation, and self-efficacy improved in the flipped educational context.

Fallah et al. (2020) examined the impact of the flipped learning approach in raising learners’ motivation and self-efficacy. They compared flipped classrooms with traditional ones. The provided out-of-class materials for learners were teacher instructional videos, pamphlets, Internet blogs, and social media, including Telegram and WhatsApp. They found out that flipped classroom technique was effective in fostering learners’ motivation and self-efficacy among learners. They asserted that students use active learning strategies such as discussion about current topics, case studies, case analysis, concept map development, problem-solving, short lectures, and small group discussions on the social media. They also added that flipped learning approach allows instructors to involve in a higher level of Bloom’s cognitive classification, including application, analysis, and combination. Moreover, they stated that learners, in using social media and educational software, have access to new mental concepts and achieve more and better skills. Finally, they argued that using social media in flipped classrooms involves learners’ different senses and makes the lesson diverse and attractive for them; hence, it seems to improve learners’ self-efficacy. Using the achievement emotion model, Zhao et al. (2021) found out that learners’ learning satisfaction, self-efficacy, and learning motivation were higher in flipped educational contexts than in traditional ones. They argued that when learners know that the instructor would observe their learning status through feedback and interaction with each other on social media, they feel pressure from their peers which ultimately fosters their learning and self-efficacy. They also stated that the use of social media in flipped classrooms will keep learners aware of their capabilities. Therefore, they can advance their ability and learn about their own self-reliance, which increases their self-efficacy.

Moreover, Latorre-Cosculluela et al.’s (2021) study revealed that learners’ self-efficacy is affected by the learning experience and the innovative educational approach like flipped learning. They justified their results according to Bandura’s social learning theory. They argued that the elements of the environment could determine learning behaviors and also influence learners’ self-efficacy beliefs. They also mentioned that a fully online learning formats like video classes, can expedite learners’ cognitive involvement, and provide guidance to interact with the learning content competently. Consequently, the chances that learners’ self-efficacy will be improved are more significant in those online flipped learning course formats. In these online learning environments, students must access the courses completely independently and plan their learning times, pace and strategies by themselves. Their study implicated that the utilizing online formats in flipped learning is a beneficial resource for developing active learning contexts in which learners can cultivate their self-efficacy.

By the same token, Luo and Gan (2022) validated flipped learning readiness factors by considering the factors, such as doing previews, in-class communication self-efficacy, positive experience, intentional behaviors, and self-directed learning. Using exploratory and confirmatory factor analysis, they found that self-efficacy is the strongest predictor of Chinese learners’ flipped learning readiness. They argued that the lack of proficiency in using social media software in flipped learning results in unproductive education, unconvincing self-efficacy, and deficient learner involvement. Their study implicated that instructors should encourage learners to reinforce their confidence and sense of achievement in language learning. In doing so, learners’ self-efficacy can be fostered, which may increase their readiness for flipped learning.

Self-efficacy, as a component of a self-regulated learning strategy, positively contributes to EFL learning (Roohani and Asiabani, 2015). According to Lai and Hwang (2016), self-regulated strategies can help learners to manage their learning, planning, monitoring, and evaluating their own learning process. They mentioned that incorporating self-regulated strategies into flipped learning through social media can foster language performance by enhancing learners’ self-efficacy. Hosseini et al. (2020) argued that integrating the self-regulated strategies under a flipped-learning context not only enables learners to use the strategy of planning, be aware of their learning process, be able to evaluate their learning, and make effective use of their study time, but also gives learners the higher level of confidence enhancing their self-efficacy. Öztürk and Çakıroğlu (2021) also explored the enhancement of learners’ language skills in a flipped classrooms designed with self-regulated learning strategies. They provided web platforms to the learners in the control group to study online to construct knowledge for the in-class sessions. However, learners in the experimental group used self-regulated learning strategies in web-based flipped classrooms by participating in forums, diary, and test modules. In these web-based modules, they employed self-regulated strategies, including time management, help-seeking, self-efficacy, organizing, rehearsing, and giving feedback. The modules comprised data about occurrences of watching videos and the number of forum messages, duration of the actions, and online test scores. Their study showed that learners’ foreign language skills are significantly affected by self-regulated learning strategies in the flipped classroom model.

Studies have shown that vicarious experience is a causal element of self-efficacy (e.g., Phan and Locke, 2015; Alrabai, 2018; Zhang and Ardasheva, 2019). Inayati and Emaliana (2017). mentioned that the incorporation of social media and technology in the educational context enriches personal as well as vicarious experiences of learners and teachers that can shape their beliefs about educational settings. Boahene et al. (2019) mentioned that sharing on social websites can expose students to new abilities resulting in more effective learning and enhancing students’ efficacy beliefs which are similar to vicarious experience. For example, research by McCoy (2010) to examine the relationship between self-efficacy and technological proficiency of students established that the use of computer at home may improve computer abilities in addition to self-efficacy. Also, mastery experience, as the primary source of self-efficacy, has been affected by social media. Siregar et al. (2020) indicated that social media as a vital learning tool in the 21st century enhances pedagogical competence, and it is effective for promoting mastery experience, translating into higher self-efficacy beliefs which are critical in enhanced performance in classroom management, instructional strategies, and student engagement activities. Bailey and Rakushin-Lee (2021) also underscored the role of social media, particularly Facebook, in enhancing learners’ master experience in educational contexts.

In addition to the effect of social media on learners’ self-efficacy, studies have highlighted the role of social media in developing other positive psychological constructs, such as well-being (Kross et al., 2021), academic engagement (Mahdiuon et al., 2020), enjoyment (Graciyal and Viswam, 2021), grit (Chua et al., 2020), resilience (Mano, 2020), and pedagogical love (Kasperski and Blau, 2020). Promoting positive traits can have substantial impacts on learning development, and many positive traits can be cultivated partially by emotional events and social influence. Today, practitioners and researchers of positive psychology may have the opportunity to design proper social media for positive development by means of observing the relationship between individual behavior and social influence. The popularity of social media can have the potential to create supportive social contexts by helping their positive development (Chua et al., 2020). The activities involved in social media, including their individual engagement and social influence, can have considerable impacts on the future development of their behavior.

## Suggestions for further research

Earlier studies have indicated that social network, such as Telegram, Facebook, Youtube, and Instagram used in flipped classrooms can develop learners’ performance and active learning by paving the way for brainstorming activities (e.g., Nugroho and Atmojo, 2020; Tsai et al., 2020). Studies have shown that social media, with interesting platforms, inspire learners to interact with peers and teachers, which in turn, enhances learners’ intention to communicate in foreign language (e.g., Khajavy et al., 2016; Mohammadi et al., 2019; Puzanov et al., 2022). Some investigations also underscored the role of Telegram in inspiring learners to engage in learning tasks, and to promote their communicative competence (Jafarigohar et al., 2019; Mohammadi et al., 2019). Studies have also shown that social media significantly affect learners’ motivation and perceived communicative competence, which influence willingness to communicate in foreign language (Nugroho, 2021; Sharma, 2019; Mulyono and Saskia, 2020; Xodabande, 2017). Moreover, investigations pinpointed the role of anxiety in willingness to communicate. In this regard, studies have shown that social media can reduce foreign language anxiety levels through fostering positive attitudes toward language learning (Sun et al., 2017; Su and Fatmawati, 2019; Mulyono and Saskia, 2020; Desta et al., 2021). The related studies have shown the positive effect of social media on learners’ self-efficacy (Zhang and Ardasheva, 2019; Fallah et al., 2020; Latorre-Cosculluela et al., 2021; Zhao et al., 2021; Luo and Gan, 2022). Investigations have indicated that learners can develop their vicarious experience and mastery experience by using social media (Alrabai, 2018; Phan and Locke, 2015; Siregar et al., 2020; Bailey and Rakushin-Lee, 2021). The capacity for teacher and peer observation in social media can increase learners’ social media. Consequently, the provision of positive psychological states through social media can increase learners’ self-reliance, which enhances their self-efficacy. Social media is useful for increasing learner engagement, which improves self-efficacy (Zhao et al., 2021).

This review probed the role of flipped learning approach in learners’ intention to communicate and self-efficacy. Studies showed that flipped classrooms, in which social media are used, increase learners’ willingness to communicate and self-efficacy. This review includes some pedagogical implications for teachers, syllabus designers, teacher educators, educational policy-makers, and advisors. In light of the related literature, teachers should be aware of using flipped teaching in their classes to increase learners’ willingness to communicate and self-efficacy. This review implicated that flipped teaching methods improve language achievement scores, and can be a means of helping students with different needs and abilities. It is significant for teachers to constantly explore new teaching methods to meet the students’ needs. Teachers can simplify the problem of learners in flipped classes by using appropriate social media to help learners become more proficient in a different context, since class time is just for practicing and problem-solving in flipped classes. However, when learners know why they learn a language, they are more cautious about the ways that facilitate this process. Teachers should provide rich opportunities for learners to in active learning while coping with their learning problems according to flipped instructions. Flipped learning also allows teachers to spend more time individually interacting with students, which creates more opportunities to check for understanding and clear up misconceptions.

A better understanding of students’ willingness to communicate in the target language may help language teachers improve the communicative language teaching approach and curriculum design to provide more communication opportunities for language learners, more importantly, encourage actual engagement in communication behaviors, and finally, facilitate second/foreign language learning and acquisition. More specifically, language instructors can enhance the level of students’ willingness to communicate through the following ways: raising students’ opportunity to talk by reducing the amount of teacher talk and allowing adequate wait-time; letting students produce language without restrictions, uncontrolled use of language; take responsibility to engage all students evenly and equally in classroom activities; videotaping themselves in the classroom, reflect on their interactional behavior to see if it has extended or limited the opportunity for the students to enter dialogs; increasing their own awareness of what interaction strategies work or do not work with specific students, and giving the instruction that lends itself to more giving and receiving of unpredictable information. Teachers can increase the amount of willingness to communicate and motivation in English classrooms by saying “Thank you” to EFL students for working hard at the end of the lesson to give positive strokes verbally or non-verbally, and encourage them. Also, the EFL students can receive different strokes by doing well or never doing well. Therefore, strokes might distinguish between successful and unsuccessful learners.

The instructors could request some foreigners, including both native and non-native speakers, to come to their classrooms to expose their students to wide variety of English dialects and accents. They might consider forming a discussion forum for all levels of students in which the students can communicate freely with foreigners without concerning about their grades. Another way for instructors to make environments for their students to interact in English would be to contact their colleagues in other countries and allow their students to interact in English *via* the Internet. A computer lab that is connected to the Internet would permit these students to have a synchronous conversations with peers in other countries. Since computer-mediated communication is believed to boost speaking, expand student motivation, and self-esteem (Compton et al., 2004), having online chats would not just enable students to communicate in English but also motivate them to learn English and enhance their self-confidence. By making environments for communication with foreigners, instructors give their students chances to share cultural knowledge with foreigners and to form realistic attitudes toward different cultures. For online and face-to-face communications, instructors may want to prepare a situation that would permit their students to share their own culture, learn about the culture of their counterparts, and gain the realization of various cultures.

Teachers have to take care of and help the learners who suffer from poor self-efficacy and help them improve in terms of self-regulation, self-esteem, and self-concept as these traits form the bases of self-efficacy (Ghonsooly and Elahi, 2010). It is believed that instructing learners on techniques to improve their self-efficacy should be given the same priority as other language skills in the EFL context. Self-efficacy could have an important role in the application and use of the approaches and methodologies in the EFL context. Instructors can use moderately-difficult activities to empower learners with low levels of self-efficacy. The activities should not be too difficult to curb learners’ self-confidence in doing tasks. Teacher support, including scaffolding, assigning sufficient time, decomposing difficult tasks into simple phases, and explicating the task in technology-supported education, are influential for enhancing leaners’ self-efficacy. This can produce an insight into reasonable challenge and equalizes the complexity of technology-supported tasks. Praising and giving feedback to learners are also crucial for improving learner self-efficacy. Moreover, teachers should not compare the performances of learners with each other. Teachers can provide learners with some strategies such as self-verbalization. For example, they can motivate learners to express the procedure of learning grammatical points or vocabulary aloud, and give feedback on their effort. Moreover, teachers can set a cooperative context, rather than a competitive one, to increase learner interaction and scaffolding, improving learner self-efficacy. They can also ask learners to write comments about their feelings and progressions in technology-supported contexts.

Moreover, teacher trainers can reach their ideal goals by considering the importance of self-efficacy, willingness to communicate, and flipped learning approach. To increase learner self-efficacy, they can implement some instructional changes in a large population of teachers by holding academic workshops. They can provide teachers with some strategies and techniques to increase learners’ willingness to communicate. In workshops, teacher trainers should suggest the consideration of learners’ experience in choosing the topic for learners. They can use survey and brainstorming techniques to collect information about topics. Moreover, the manner of error correction and the grouping of learners should be provided to instructors to boost learners’ willingness to communicate. Teachers can group learners based on learners’ topics of interest or their level of language ability.

Educational policy-makers should hire experienced teachers, as the instructive experience can be an important issue for increasing self-efficacy and willingness to communicate among learners. They can ask teachers to do their best within varied educational contexts. They must build up teaching effectiveness by providing contexts for observations of other teachers’ activities and mastery experiences to increase learners’ engagement in language production in particular ranges of instruction. They should also provide critical thinking, creativeness, and motivation to the education in classrooms which encourage self-efficacy. The importance of self-efficacy, and willingness to communicate can motivate advisors to expand their horizons to identify learners’ sources of self-efficacy, and to probe the reasons for increasing oral communication skill.

Other emotional constructs, including grit, foreign language enjoyment, learner engagement, pedagogical love, and resilience, can be investigated in digital-based flipped learning classrooms. It would also prove productive to inspect teachers’ perceptions of the communication behaviors of their learners in flipped and traditional classrooms. Moreover, the effect of different social networks on learners’ self-efficacy and willingness to communicate in a foreign language can be studied in the future. Moreover, the relationship between different language skills and other positive psychological constructs, such as academic engagement, well-being, enjoyment, and resilience in flipped classrooms can be investigated in the future. Future research can be conducted about the relationship between teaching style, and learners’ willingness to communicate in flipped classrooms is a big issue to explore. Moreover, the negative emotional states, such as foreign language anxiety, apprehension, boredom, and burnout, can be studied in traditional, blended, and flipped classrooms.

## Author contributions

XF the sole author, independently drafted this manuscript, and submitted to this journal for consideration of potential publication.
